# Hepatitis B Recurrence After Liver Transplantation: A Single Center Experiences and Review the Literature

**DOI:** 10.5812/hepatmon.6609

**Published:** 2013-01-01

**Authors:** Seyed Mohsen Dehghani, Seyed Ali Reza Taghavi, Bita Geramizadeh, Saman Nikeghbalian, Nima Derakhshan, Abdorrasoul Malekpour, Seyed Ali Malek-Hosseini

**Affiliations:** 1 Shiraz Transplant Research Center, Nemazee Hospital, Shiraz University of Medical Sciences, Shiraz, IR Iran; 2 Gastroenterohepatology Research Center, Nemazee Hospital, Shiraz University of Medical Sciences, Shiraz, IR Iran

**Keywords:** Hepatitis B Virus, Liver Transplant, Immunosuppression, Recurrence

## Abstract

**Background:**

Despite the advances in the treatment of chronic hepatitis B virus (HBV) infection, liver transplantation (LT) remains the only hope for many patients with end-stage liver diseases resulting from HBV.

**Objectives:**

The aim of this study was to investigate the rate of HBV recurrence in cases that had undergone LT due to the HBV related liver cirrhosis.

**Patients and Methods:**

Forty-nine patients who underwent LT due to HBV related cirrhosis since 2001 to 2009 in Shiraz Organ Transplantation Center were enrolled in the present study. They were asked to complete the planned questionnaire and also to sign the informed consent in order to take part in this study. Post-transplant prophylaxis protocol against HBV recurrence was based on a hundred milligrams of lamivudine daily plus intramuscular injections of hepatitis B immune globulin (HBIG) with appropriate dosage to keep anti-HBs antibody titer above 300 IU/L and 100 IU/L in the first six months and afterwards, respectively. Blood samples were obtained and checked for HBsAg, HBeAg, and the titers of Anti -HBsAb as well as Anti- HBeAb with ELISA. A quantitative HBV DNA assay was also done on all samples (GENE-RAD® Real-time PCR).

**Results:**

There were 91.8% males and 8.2% females enrolled in the study. The duration of post-transplant prophylaxis ranged from 3 months to 8 years (mean 18.9 ± 19.3 months). HBsAg and HBeAg were positive in 24.5% and 2% of cases, respectively. Real-time PCR for HBV DNA were zero copies/mL in 91.8% of patients, none of which represented a positive value for HBV recurrence (Positive > 10,000 copies/mL). The mean Anti-HBs Ab titer was 231.7 ± 135.9 IU/L; it was above 100 IU/L in 71.4% of patients. Thirty-seven (75.5%) of the patients were taking tacrolimus plus mycophenolate mofetil, 6 (12.2%) were on cyclosporine plus mycophenolate mofetil, and 6 (12.2%) were taking sirolimus plus mycophenolate mofetil. HBsAg was detectable in seven patients taking tacrolimus plus mycophenolate mofetil (18.9%), in four patients taking cyclosporine plus mycophenolate mofetil (66.7%), and in one patient among the six who were taking sirolimus plus mycophenolate mofetil (16.7%). There was no significant statistical correlation between the presence of a positive value for HBsAg and the immunosuppression regimen or Anti HBsAb titer (P ˃ 0.05). Presence of a positive value for HBsAg was not predictive of a positive HBV DNA or its level in blood (P ˃ 0.05).

**Conclusions:**

Post-transplant HBV prophylaxis with lamivudine and intramuscular HBIG with appropriate dosage to keep anti-HBs antibody titer above 300 IU/L in the first six months and above 100 IU/L afterwards is effective for prevention of HBV recurrence after LT.

## 1. Background

Hepatitis B virus (HBV) is a double-stranded DNA virus belonging to the family of hepadnaviridae (1). Chronic hepatitis B or C causes severe liver diseases, such as liver cirrhosis and hepatocellular carcinoma (HCC) (2). The main indications for liver transplantation (LT) in the Western Europe and the United States are both HBV and hepatitis C virus (HCV) related cirrhosis, especially HCV infection (3, 4). Recurrence of HBV or HCV infection after LT plays a key role in the outcome of LT regarding both the patient and the graft survival (5, 6). It seems that recurrence of viral hepatitis is associated with allograft dysfunctions, cirrhosis of the allograft, and graft failure as major complications. Nowadays, overall survival of patients transplanted for HBV related cirrhosis exceeds 85 percent in one year and 75 percent in five years (7-9). Over the last 10–20 years, the results of HBV related LT were reported to be as good as or even better than LT for other diseases (7, 8). The high rate of HBV recurrence following LT was probably due to the enhanced virus replication resulting from immunosuppression. Nevertheless, the number of reports on this issue was limited, especially regarding the types of immunosuppressive regimens.

## 2. Objectives

The present study aimed to report the rate of HBV recurrence in our cases that had undergone LT due to the HBV related liver cirrhosis from 2001 to 2009. It also aimed to determine whether there is a difference between the rates of recurrence in patients taking different immunosuppressive regimens.

## 3. Patients and Methods

All forty-nine patients who underwent LT due to HBV related cirrhosis since 2001 to 2009 in Shiraz Organ Transplantation Center affiliated with Shiraz University of Medical Sciences were enrolled in this study. The exclusion criteria of the study were undergoing LT before 2001, after 2009, or due to other diseases. Also, the patients who died after transplantation were excluded from this study. Demographic data such as the age at the time of diagnosis, sex, current age, lag periods between the diagnosis and transplantation, duration of follow up, pre-transplant antiviral medications, post-transplant medications for HBV recurrence prophylaxis, and the immunosuppressive regimens were obtained from the patients’ hospital records. Routine post-transplant prophylactic protocol against HBV recurrence in our center was based on a hundred milligrams of lamivudine daily plus intramuscular injections of hepatitis B immune globulin (HBIG) with appropriate dosage to keep anti-HBs antibody titer above 300 IU/L in the first six months and above 100 IU/L afterwards. The patients were contacted from all over Iran, since currently, this center is the only active LT center in Iran. Then, they were asked to complete the questionnaire and sign the informed consent for participation in this study. Upon their referral for follow up visits, blood samples were collected to check HBsAg, HBsAb, HBeAg, HBeAb (ELISA), and HBV DNA levels (GENE-RAD® Real-time PCR). The study was approved by Local Ethics Committee of Shiraz University of Medical Sciences.

### 3.1. Serological Measurements

HBsAb and HBeAb titers were assessed using standard ELISA methods. Also, HBsAg and HBeAg were detected using Counter Current Immunoelectrophoresis (CCIEP) method. Quantitative real-time reverse transcription-polymerase chain reaction (qPCR) was used to detect HBV recurrence. Real-time quantitative PCR was carried out using the Rotorgene 6000 software. In addition, GENE-RAD® Real-time PCR kits were used for HBV. Primers HBV-Taq 1 (5′- CAA CCT CCA ATC ACT CAC CAA C -3′) and HBV-Taq 2 (5′- ATA TGA TAA AAC GCC GCA GAC AC -3′), and probe HBV-P (5′- TCC TCC AAT TTG TCC TGG TTA TCG CT-3′), whose 5′-labelled with FAM and VIC, respectively, and 3′-labelled with TAMRA, were used to detect the amount of HBV DNA (Applied Biosystems, Warrington, UK) (10).

### 3.2. Statistical Analysis

All statistical analysis was performed using SPSS statistical analysis software (Version 16). T-Fisher's exact and Chi-Square were used to analyze obtained data; P < 0.05 was considered as statistically significant.

## 4. Results

Among forty-nine selected patients, forty-five (91.8%) were males and four (8.2%) were females. The lag period between the diagnosis of HBV cirrhosis and LT ranged from seven months to 27 years (6.7 ± 5.9 years). The duration of pre-transplant treatment with lamivudine (plus adefovir in three cases, and plus interferon in 5 cases) ranged from two months to 10 years (3.2 ± 2.4 years).The total duration of post-transplant prophylaxis with lamivudine and HBIG ranged from 3 months to 8 years (mean 18.9 ± 19.3 months). None of the patients had any clinical or biochemical evidences of liver diseases at the time of the trial. HBsAg was positive in twelve (24.5%) and negative in thirty-seven patients (75.5%). HBeAg was positive in only one (2%) and negative in the rest of patients. Real-time PCR for HBV DNA was zero copy/mL in forty-five patients and detectable in four (2 copies/mL in two patients, 18 copies/mL in one, and 4072 copies/mL in one, none which represented a positive value for HBV recurrence, Positive > 10,000 copies/mL). HBsAb titer ranging from 0.2 to 360.5 IU/L (mean 231.7 ± 135.9 IU/L) was above 100 IU/L in 35 patients (71.4%), and below 100 IU/L in 14 patients (28.6%). Twenty-six patients (53.1%) had HBsAb titer above 300 IU/L, nine patients between 100 and 300 IU/L (18.4%), ten patients between 10 and 100 IU/L (20.4%), and only four patients under 10 IU/L (8.2%). HBeAb titer ranged from 0.35 to 10.25 IU/L (mean 3.53 ± 3.28 IU/L). Moreover, twenty-nine patients (59.2%) had high titers of HBeAb, 19 patients (38.8%) had a negative value for HBeAb, and one patient had an equivocal value. In the present study, the patients were on three different immunosuppressive regimens; thirty-seven were taking tacrolimus plus mycophenolate mofetil (75.5%), six were on cyclosporine plus mycophenolate mofetil (12.2%), and six were taking sirolimus plus mycophenolate mofetil (12.2%). Prednisolone was tapered and discontinued during 1-3 months after LT in all patients. HBsAg was positive in 7 patients taking tacrolimus plus mycophenolate mofetil (18.9%), 4 patients taking cyclosporine plus mycophenolate mofetil (66.7%), and one patient among the six who were taking sirolimus plus mycophenolate mofetil (16.7%). There was, however, no significant statistical correlation between the type of the immunosuppression and HBsAg positivity (P ˃ 0.05). There was no significant correlation between the rate of HBsAg seropositivity and age at diagnosis, lag time between end-stage liver disease and LT, duration of taking antiviral agents before the transplantation, the duration of taking HBIG, and antiviral agents after the transplantation (P ˃ 0.05). HBsAb titer also did not have any correlation with age at diagnosis, lag time between end-stage liver disease and LT, period of taking pre-transplantation medications, and duration of taking post-transplantation medications (p ˃ 0.05). HBsAb titer was above 100 IU/L in 28 out of 37 patients taking tacrolimus plus mycophenolate mofetil (75.7%), 4 out of 6 patients taking cyclosporine plus mycophenolate mofetil (66.7%), and 3 out of 6 patients taking sirolimus plus mycophenolate mofetil (50%). In addition, twenty-two out of 37 patients (59.5%) in the first group, 3 out of 6 patients (50%) in the second group, and 1 out of 6 patients (16.7%) in the third group had HBsAb titers above 300 IU/L. HBeAg was positive in only one and HBV DNA titer was negative in all patients; therefore, the statistical analysis for finding the correlation between the above mentioned factors was impossible.

## 5. Discussion

The spectrum of clinical HBV infection during the acute phase ranges from subclinical or anicteric hepatitis to icteric hepatitis and, in some cases, fulminant hepatitis. During the chronic phase, manifestations range from an asymptomatic carrier state to chronic hepatitis, cirrhosis, and HCC (11). Most of the LT performed in our center for chronic viral hepatitis were on HBV induced cirrhosis, rather than HCV cirrhosis, which may be due to the lower rate of infection with HCV in our population compared to the reported rates from other centers. The initial poor results of LT in the patients suffering from chronic hepatitis B in the 1980s were due to recurrence rates approaching 80 to 100 percent (12-14). Recurrence of HBV or HCV infection plays a key role for the outcome after LT in the patients with viral hepatitis. Allograft dysfunctions, cirrhosis of the allograft, and graft failure are major complications of HBV or HCV recurrence (3). The high rate of recurrence of HBV infection after LT is probably due to enhanced virus replication as a result of immunosuppression as well as direct stimulatory effects of glucocorticoids on glucocorticoid-responsive enhancer region of the HBV genome (15, 16). Consequently, it was proposed that corticosteroids should be rapidly removed from immunosuppressive regimen to minimize the risk of HBV recurrence. So far, although many centers practice the early withdrawal of steroids in HBV patients after LT, there are no studies to proof the better outcome under this regimen (3). Extra-hepatic reservoirs of HBV, such as peripheral blood mononuclear cells, spleen, and other organs, may also play a role in graft reinfection (17). HBV recurrence is diagnosed by reappearance of HBsAg in the serum and most of patients are HBeAg positive and have a high HBV DNA titer. Recurrence of HBV after LT is almost always accompanied by recurrent liver disease which is often severe and rapidly progressive. It is associated with several factors such as pre- and post-transplant medications. High-risk patients include those with cirrhosis who are either HBeAg positive or HBeAg negative but have high serum HBV DNA levels, as well as those with antiviral drug-resistance prior to the transplantation (17-19). On the other hand, low-risk patients are the ones with fulminant HBV, co-infection with HDV, and cirrhotic patients who are HBeAg negative with low serum HBV DNA levels at the time of transplantation (9). Since late 1980s, the introduction of effective measures for preventing and treating recurrence has significantly improved the outcome of LT using strategies involving HBIG and, subsequently nucleoside (tide) analogues (7, 20, 21). Prevention of HBV recurrence includes antiviral therapy before the transplantation and combination of antiviral therapy and HBIG after transplantation. This strategy has led to a reduction in HBV recurrence rate to less than 10 percent (3, 22). Passive immunoprophylaxis with HBIG was first introduced in early 1990s and dramatically reduced the rate of recurrence after LT. Samuel et al. showed significant reduction of HBV recurrence rates as well as survival improvement in hepatitis B patients receiving long-term treatment with HBIG after LT (20). The results have been confirmed in many studies, thereafter (23). The short term application, however, did not improve the outcome with constant recurrence rates after LT (3). Nevertheless, HBV recurrence was detected in 15–50% of patients receiving indefinite HBIG prophylaxis. HBV recurrence under ongoing HBIG prophylaxis can be caused by escape mutations with reduced affinity to monoclonal or polyclonal anti-HBs antibodies (24). High dose HBIG prophylaxis with anti-HBsAb titers > 500 IU/L can reduce the development of HBsAg escape mutants; however, it could not completely prevent the occurrence of mutations. Besides, 10–20% of patients show HBV recurrence even under high dose HBIG application. Therefore, due to additional therapeutic opportunities HBIG should not be used as monoprophylaxis for preventing the HBV recurrence (3). Inhibition of HBV replication is another approach to prevent HBV recurrence of the allograft. Lamivudine was the first inhibitor of HBV replication approved for treating chronic hepatitis B. It is a nucleoside analogue which competitively inhibits the reverse transcriptase and termination of proviral DNA chain extension. While the short-term results of lamivudine monoprophylaxis and administered pre- as well as post-LT showed excellent outcomes with a 1-year recurrence rate of 10% and seroconversion to HBsAg negativity in 100%, the recurrence rates of 50% were observed in long-term follow up (25). Lamivudine resistant mutants, mainly the mutations within the tyrosine-methionine-aspartate-aspartate (YMDD), and motif of HBV DNA polymerase lead to those high recurrence rates in the long-term follow up. In addition, immunosuppression has a great influence on mutation rate. Lamivudine resistance was detected in 15% of the immunocompetent patients within the first treatment-year in comparison to 45% of immunosuppressed patients (26, 27). Monoprophylaxis with lamivudine is only partially effective for preventing HBV recurrence. Moreover, occurrence of YMDD-mutant strains leads to HBV recurrence under ongoing therapy with lamivudine. As a result, monoprophylaxis with lamivudine cannot be recommended as a standard regimen, since YMDD mutants occur more rapidly in immunosuppressed patients (3). Because both HBIG and lamivudine in a monoprophylactic approach show higher recurrence rates in comparison to prophylaxis with a combination of HBIG and lamivudine, most centers like ours use the combination prophylaxis as a standard regimen. Mean recurrence rates of about 5% (0-10%) in combination prophylaxis are lower compared to either HBIG or lamivudine monoprophylaxis. These results have been confirmed in several studies that consistent with our results (28). Mostly, lamivudine therapy starts in the pre-LT settings combined with HBIG at LT (5). In a clinical trial with 29 patients, Buti et al. (29) reported successful discontinuation of HBIG after one month combined prophylaxis. It seems to be feasible to continue with lamivudine monoprophylaxis combined with prophylaxis with HBIG after LT in low risk patients by carefully monitoring of HBV DNA (3). Another promising approach to reduce HBIG dosages was switching the mode of application from intravenous to intramuscular (30-32). However, rapid developments of resistant mutants with long-term lamivudine prophylaxis as well as moderate reduction in HBV DNA levels at best and ineffectively in patients with prior lamivudine resistance are potential problems with lamivudine mono prophylaxis (33, 34). However, the problem could be solved by using adefovir which is a new drug with activity against the lamivudine resistant mutants (35). Adefovir is a nucleotide analogue that acts as a chain terminator and is supposed to stimulate the natural killer cells (36). In addition, adefovir shows a very low rate of drug resistance (3). Compared with lamivudine, adefovir-resistant mutants occur more slowly. The incidence HBV recurrence in the non-transplant settings was reported as fewer than 4% after 2-year adefovir treatment, while, it was increased to more than 20% after 4 years. Fortunately, the mutant strains showed all clinical responses to lamivudine (37). In addition to lamivudine and adefovir, there are several new antiviral drugs with high activity against HBV, such as the nucleotide analogue tenofovir, the nucleoside analogues entecavir, and telbivudine. Indeed, their anti-HBV activity seems to be higher compared to lamivudine or adefovir. In non-transplant settings, entecavir and telbivudine showed a high efficacy in suppressing viral replication (38, 39). Furthermore, tenofovir is highly effective in chronic HBV infections presenting YMDD-mutants (40). These drugs are under clinical investigation in non-transplant and partially in transplant settings. Moreover, they may play a role in preventing HBV recurrence after LT in the future (3). Our current prophylaxis protocol is daily lamivudine 100 mg pre-transplant and daily 100 mg plus intramuscular HBIG. Post-transplant HBIG intramuscular injections are adjusted to keep anti-HBs antibody titer over 300 IU/L in the first 6-months and over 100 IU/L afterwards. Patients with preexisting lamivudine resistance took adefovir. According to the results of the present study, with this policy we did not have any significant HBV recurrence in our patients. All those with positive HBsAg had low HBV DNA levels indicating a non-replicative status and most likely clinically non-significant infection. Cyclosporine, tacrolimus, and prednisolone, which are the most widely used immunosuppressive agents, have proved to be able to prevent rejection. Recently, new immunosuppressant agents, such as sirolimus, mycophenolate mofetil, and anti-interleukin-2 receptor monoclonal antibodies have been available. In addition, other new drugs such as FTY 720, FK 778, anti-CD20, anti-CD40, and anti-CH52 monoclonal antibodies are now being evaluated in clinical trials(41, 42). The immunosuppression protocol of this center is mycophenolate mofetil plus one of the three following medications: cyclosporine, tacrolimus, and sirolimus. In this study, we did not find any significant correlation between the type of immunosuppressive regimens and HBsAg seropositivity. Ying et al (43) reported that mycophenolic acid and ribavirin, both inhibitors of IMP-DH, potentiated the anti-HBV activity of guanine-based nucleoside analogues in vitro. In addition, some studies found that mycophenolic acid could inhibit HBV replication in HepG2-2-15 cell and human hepatocyte (44, 45). Our results may be in some part due to our immunosuppressant regimen that included mycophenolate mofetil in all patients. On the contrary, several studies have demonstrated that long-term use of immunosuppressant drugs after LT might promote the vigorous replication of HBV directly or through inhibiting the immune system. Immunosuppressant agents may impair T cell function and, as a result, reduce the immune-mediated hepatocytolysis and virus clearance. In addition, corticosteroid may activate the glucocorticoid responsive element in HBV genome to enhance HBV replication and gene expression (46, 47). Thus, the level of immunosuppression should be determined considering the balance between HBV recurrence in the graft and the risk of rejection (48). Tisone et al. (49) believed that steroid-free immunosuppression in LT patients was safe and effective. Further dual-random and controlled clinical trials are needed to evaluate the influence of immunosuppressant drugs on HBV recurrence. Limitation of this study was the lack of any data about HBeAg status and HBV DNA titers of patients before LT; so we could not determine if our cases were high risk or low risk for HBV recurrence after LT. The low number of patients preventing statistical comparison between some variables also can be considered as a limitation. We conclude that post-transplant HBV prophylaxis with lamivudine and intramuscular HBIG with appropriate dosage to keep anti-HBs antibody titer above 300 IU/L in the first six months and above 100 IU/L afterwards are effective for prevention of HBV recurrence after LT.
